# Aerosol Transmission of *Aspergillus fumigatus* in Cystic Fibrosis Patients in the Netherlands

**DOI:** 10.3201/eid2504.181110

**Published:** 2019-04

**Authors:** Tobias G.P. Engel, Ellen Erren, Koen S.J. Vanden Driessche, Willem J.G. Melchers, Monique H. Reijers, Peter Merkus, Paul E. Verweij

**Affiliations:** Radboud University Medical Center, Nijmegen, the Netherlands (T.G.P. Engel, E. Erren, K.S.J. Vanden Driessche, W.J.G. Melchers, M.H. Reijers, P. Merkus, P.E. Verweij);; Center of Expertise in Mycology Radboud UMC/CWZ, Nijmegen (T.G.P. Engel, W.J.G. Melchers, M.H. Reijers, P.E. Verweij)

**Keywords:** cystic fibrosis, aspergillus infection, fungi, infection prevention, cough, aerosol, patient-to-patient transmission, cough plate, *Aspergillus fumigatus*, sputum culture, respiratory infections, saprophytic mold, airway colonization, chronic infection, the Netherlands

## Abstract

We collected sputum samples and cough plates from 15 cystic fibrosis patients in the Netherlands who were colonized with *Aspergillus fumigatus*; we recovered *A. fumigatus* of the same genotype in cough aerosols and sputum samples from 2 patients. The belief that transmission of *A. fumigatus* from cystic fibrosis patients does not occur should be reconsidered.

Progressive lung injury in cystic fibrosis (CF) patients can lead to chronic colonization with bacteria and fungi (*1*,*2*). Different routes of patient-to-patient transmission have been identified for various microorganisms (*3*). For saprophytic molds, such as *Aspergillus fumigatus*, exposure to aerosolized conidia in the environment is believed to be the primary route leading to colonization of the airways (*3*). Secretion of these fungi from the human lung into the environment is thought not to occur, and the general view is that humans are dead-end hosts of filamentous fungi. However, some reports have provided information suggesting that this belief might need to be revised (*4*). We set out to examine whether aerosol formation of *A. fumigatus* occurs in CF patients during coughing.

## The Study

During 2017–2018, we invited 15 adult CF patients in the Netherlands who were colonized with *A. fumigatus* to participate in a cough plate experiment. We defined *A. fumigatus* colonization as the recovery of *A. fumigatus* from >50% of sputum samples collected over the course of the 2 previous years. To enable genetic comparisons among study samples, we stored cultured fungal isolates from included patients in the Radboud University Medical Center Medical Microbiology Department (Nijmegen, the Netherlands) fungal species bank. We performed sample collection during 2 routine quarterly visits before or after routinely performed spirometry. We instructed participants to take a maximal inspiration and cough twice on each of 2 different agar plates, Sabouraud dextrose agar and Columbia blood agar, held at a 5-cm distance from the participant’s mouth. In addition, we collected a sputum sample on the same day or alternatively within a month if the participant was unable to produce sputum during the visit. We incubated Sabouraud dextrose agar cough plates at 28°C and Columbia blood agar cough plates at 36°C for 3 weeks and inspected daily for bacterial and fungal growth. We processed sputum samples according to CF guidelines (*3*). We collected individual CFUs (up to a maximum of 20 CFUs per sample) from cough plates and sputum cultures and genotyped *A. fumigatus* CFUs using microsatellite genotyping (*5*). We considered the detection of identical *A. fumigatus* genotypes from cough plates and sputum samples as proof of aerosolization of *A. fumigatus* from humans. We genotyped up to 6 sputum cultures of stored *A. fumigatus* isolates from each participant.

This study was reviewed by the institutional review board, which considered the study exempt from further institutional review board oversight in accordance with the law in the Netherlands on research with humans. All participants provided informed consent.

We cultured *A. fumigatus* from 18 (60%) sputum samples collected from 11 different participants (Table). *A. fumigatus* was also recovered from 3 (17%) of the 18 corresponding cough plate samples; these 3 samples were from 2 different participants. In both cases, cough plates had been acquired after the participant had undergone spirometry. Genotyping of the *A. fumigatus* isolates from sputum samples and cough plates from both participants showed identical genotypes. One participant was colonized with at least 10 different *A. fumigatus* genotypes (Figure). In this participant, the genotype recovered from the cough plate was not found in the sputum sample collected at the same visit but in the sputum sample recovered at the next visit. The second participant was colonized with a single genotype, which was recovered from multiple cough plates and sputum samples.

**Table:** Characteristics of and growth of *Aspergillus fumigatus*, *Pseudomonas aeruginosa*, *Staphylococcus aureus*, and *Stenotrophomonas maltophilia* in sputum samples and on cough plates from 15 cystic fibrosis patients, the Netherlands, 2017–2018*

Pt. no.	Age, y/sex	FEV1, L (% predicted)	Peak flow, L/s	AB/AF use	Growth on first/second cough plates					Cultured from first/second sputum sample			
*A. fumigatus*	*P. aeruginosa*	*S. aureus*	*S. maltophilia*	*A. fumigatus*	*P. aeruginosa*	*S. aureus*	*S. maltophilia*
1	23/F	2.7 (85)	7.9	Yes/no	–/–	+/–	–/–	–/–		–/–	+/+	+/+	+/–
2	20/F	4.0 (101)	8.6	Yes/no	–/–	–/–	–/–	–/+		–/ND	–/ND	+/ND	+/ND
3	22/F	2.3 (74)	7.9	Yes/no	–/–	–/–	–/+	–/–		+/+	–/–	+/+	–/–
4	20/M	3.2 (61)	7.4	Yes/no	+/–	–/–	–/–	–/–		+/+	–/–	+/+	–/–
5	49/F	2.3 (74)	7.7	No/no	–/–	–/–	–/–	–/–		+/–	–/–	+/+	–/–
6	43/F	2.7 (82)	7.2	Yes/no	–/–	–/–	–/–	–/–		+/+	+/+	–/–	–/–
7	26/M	4.6 (106)	10.9	Yes/no	–/–	–/–	+/+	–/–		+/ND	–/ND	+/ND	–/ND
8	39/F	1.0 (33)	5.7	Yes/no	–/–	–/–	–/+	–/–		+/+	–/–	–/–	+/+
9	49/M	1.2 (31)	3.4	Yes/no	–/–	+/+	–/–	–/–		+/+	+/+	–/–	–/–
10	22/M	2.0 (41)	7.8	Yes/no	–/–	+/+	–/–	–/–		+/–	+/+	+/+	–/–
11	58/F	2.2 (79)	5.1	Yes/no	–/–	–/–	–/–	–/–		–/–	+/+	+/–	–/–
12	31/M	2.0 (41)	5.3	Yes/no	–/–	+/+	+/+	–/–		–/+	–/+	–/+	–/–
13	51/F	0.7 (26)	3.5	Yes/no	–/–	+/+	–/–	–/–		–/–	+/+	+/+	–/–
14	30/M	2.5 (50)	8.7	Yes/no	–/–	+/+	–/–	–/–		+/+	+/+	–/–	–/+
15	27/F	3.8 (102)	8.4	Yes/no	+/+	–/–	–/–	–/–		+/+	–/–	–/–	–/–

*AB, antibiotic; AF, antifungal; FEV1, forced expiratory volume in 1 second; ND, not done; Pt, patient; +, positive; –, negative.

**Figure:**
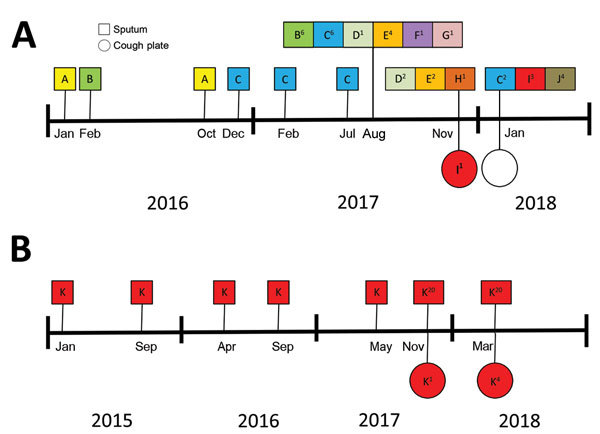
Genotyping results of *Aspergillus fumigatus* isolates in sputum cultures and on cough plates obtained from 2 participants with cystic fibrosis demonstrating aerosol formation of *A. fumigatus*, the Netherlands, 2015–2018. For samples collected after August 2017, a maximum of 20 isolates per sputum culture were saved. For samples collected earlier, only 1 sputum sample isolate was saved. Genotypes of the isolates collected from patient 4 (A) and 15 (B) are indicated. The superscript number indicates the number of isolates of that same genotype cultured from the collected sample.

We also assessed for bacterial species present on cough plates and in sputum samples to enable comparison of transmission frequencies among pathogens. The cough secretion frequency for *Pseudomonas aeruginosa* was 67% (10/15) and for *Staphylococcus aureus* was 19% (3/16) (Table). No growth of *Stenotrophomonas maltophilia* on cough plates was observed.

Our study demonstrates that *A. fumigatus* can be recovered from cough aerosols from colonized CF patients with a similar frequency as *S. aureus.* This aerosolization is strikingly occurring in CF outpatients without any cavitary lesions or other serious complications. Cavitary lesions can facilitate sporulation of *A. fumigatus* inside patient lungs and thereby aids in fungal secretion.

By continuously monitoring indoor airborne fungal contamination with electrostatic dustfall collectors, Lemaire et al. identified patient airways as the source of *A. fumigatus* contamination in an intensive care unit (*4*). Microsatellite genotyping showed that the airborne *A. fumigatus* and isolates from the patient’s respiratory samples were identical. That observation and our study results indicate that the current consensus that transmission of *A. fumigatus* from colonized or infected patients does not occur should be reconsidered.

Although CF patients are typically colonized with unique *A. fumigatus* genotypes, several studies report the recovery of identical *A. fumigatus* genotypes from different CF patients (*6*). These genotypes could not be linked to a common environmental source and thus remained unexplained, but identical genotypes in different patients might point toward patient-to-patient transmission (*6*). Patient-derived azole resistance mutations have been reported in CF patient (*7*) and environmental (*8*) isolates. The mechanism for acquisition of azole resistance is unknown for environmental isolates, but resistance might have developed in isolates during human infection or colonization and then subsequently spread into the environment. Our observation of *A. fumigatus* in cough aerosols suggests droplet or airborne transmission as potential routes of patient-to-patient transmission. Traits such as azole resistance might spread through cough aerosols from patient to patient or from patient to environment. Both participants with positive cough plates had undergone spirometry before coughing, indicating that maximal inspiration and expiration might facilitate the release of *A. fumigatus*.

Aerosolized *A. fumigatus* conidia from environmental sources could represent a greater and more continuous burden for CF patients than potentially aerosolized conidia from patients. However, some studies suggest that *A. fumigatus* in chronically infected patients undergoes an evolutionary trajectory resulting in strains with specific traits that are better adapted to the lung environment (*9*,*10*). Endogenous and exogenous stress factors, such as host immunity or exposure to antifungal azoles, might result in the selection of traits that are better able to resist these stress factors (*11*). Isolates that are better adapted to the lung environment might have a greater propensity than environmental isolates to successfully colonize and persist in the airways of CF patients. In CF, 42% of azole resistance is derived through in-host selection and is thought to be associated with prolonged (prophylactic) use of anti-*Aspergillus* azoles (*12*,*13*). Passaging through the lungs of CF patients conceivably could provide *A. fumigatus* isolates the opportunity to acquire specific traits that increase their ability to survive in different environments. Evolution experiments have confirmed the potential of *A. fumigatus* to rapidly adapt to various environments (*9*).

## Conclusions

In summary, our results show that *A. fumigatus* can be recovered from cough aerosols of colonized CF patients. These findings underscore the need for additional studies to further elaborate transmission dynamics of *A. fumigatus*, evaluate if patient-to-patient transmission occurs, and determine if additional infection prevention measures are required.
